# Monkeypox Detection Using CNN with Transfer Learning

**DOI:** 10.3390/s23041783

**Published:** 2023-02-05

**Authors:** Murat Altun, Hüseyin Gürüler, Osman Özkaraca, Faheem Khan, Jawad Khan, Youngmoon Lee

**Affiliations:** 1 Department of Information Systems Engineering, Faculty of Technology, Mugla Sitki Kocman University, Mugla 48000, Turkey; 2 Department of Computer Engineering, Gachon University, Seongnam-si 13120, Republic of Korea; 3 Department of Robotics, Hanyang University, Ansan 15588, Republic of Korea

**Keywords:** monkeypox detection, healthcare, epidemics, deep learning, transfer learning, image processing

## Abstract

Monkeypox disease is caused by a virus that causes lesions on the skin and has been observed on the African continent in the past years. The fatal consequences caused by virus infections after the COVID pandemic have caused fear and panic among the public. As a result of COVID reaching the pandemic dimension, the development and implementation of rapid detection methods have become important. In this context, our study aims to detect monkeypox disease in case of a possible pandemic through skin lesions with deep-learning methods in a fast and safe way. Deep-learning methods were supported with transfer learning tools and hyperparameter optimization was provided. In the CNN structure, a hybrid function learning model was developed by customizing the transfer learning model together with hyperparameters. Implemented on the custom model MobileNetV3-s, EfficientNetV2, ResNET50, Vgg19, DenseNet121, and Xception models. In our study, AUC, accuracy, recall, loss, and F1-score metrics were used for evaluation and comparison. The optimized hybrid MobileNetV3-s model achieved the best score, with an average F1-score of 0.98, AUC of 0.99, accuracy of 0.96, and recall of 0.97. In this study, convolutional neural networks were used in conjunction with optimization of hyperparameters and a customized hybrid function transfer learning model to achieve striking results when a custom CNN model was developed. The custom CNN model design we have proposed is proof of how successfully and quickly the deep learning methods can achieve results in classification and discrimination.

## 1. Introduction

Monkeypox is a disease caused by the monkeypox virus, which belongs to the family of viruses called Poxviridae and the genus orthopoxviral. Variola virus, which is another virus in the Poxviridae family, causes smallpox. The cowpox virus causes bovine smallpox, and the Vaccinia virus is used in the production of smallpox vaccines. Although the name of the disease is monkeypox, the virus is actually of rodent origin. The virus, which was detected in 1958 as a result of two different outbreaks with symptoms similar to smallpox in monkey colonies kept for research, was therefore called monkeypox. In humans, an infection caused by the monkeypox virus was first observed in 1970. From those years to the present day, monkeypox disease has been seen as a rare case on the African continent. Monkeypox, which has been seen for many years in West and Central Africa, where tropical rainforests are abundant and limited to this region, rarely reached different parts of the world, with transmission caused by animals exported from the region. However, in the recent period, the disease has become more widespread than in the past and has been diagnosed in different people from different regions. Monkeypox cases, which are seen as a possible new virus outbreak due to the devastating effects of the COVID-19 pandemic on the world, have started to be followed in detail, even though they are not yet at the pandemic stage and show an epidemic spread [1].

In monkeypox disease, rashes appear on the skin over a range of 1–5 days. Often, the first rashes are observed in the facial area and then spread to other parts of the body. In some patients, lesions can also be seen in the genital area, eyes, and intraoral mucosa. This disease can be confused because of its similarity to chickenpox rashes. While these rashes are initially water-filled blisters, over time they turn into crusty spots and begin to heal. In some patients, the lesions involve hundreds of bumps that spread throughout the body, while in other patients fewer blisters occur. In intensive cases, the lesions may merge and cause large-scale rashes on the skin surface. Depending on the severity of the disease, the rashes usually disappear completely over a range of 2 to 4 weeks and the disease heals.

When the latest technologies in the field of image processing and classification are examined, we see that convolutional neural networks (CNNs) are frequently used in academic studies in the field of deep learning. CNN is a deep learning model that typically takes image data as input. It captures future inferences from the image with different operations and classifies them. The first CNN structure is the LeNet architecture, which was introduced by Yann LeCun in 1988 and continued to be improved until 1998 [2]. CNN algorithms are applied in many different fields, such as natural language processing (NLP) and biomedicine, especially in the field of image and sound processing. Especially in the field of image processing, the best (state-of-the-art) results have been obtained. On the MNIST dataset, the authors [3] managed to reduce the error rate to 0.23% with CNN. Section 3 provides detailed information about the CNN working structure.

The most prominent feature of monkeypox disease is its lesions on the skin. Rapid differentiation of skin lesions resulting from this disease from other lesion illnesses is important for early initiation of treatment. The skin lesions of monkeypox should be differentiated from other similar skin lesion diseases by mobile devices, and the risk of transmission should be minimized. An image taken with the help of a camera on a mobile device and tested on the trained TFLite model obtained by the transfer learning method will give the end user positive or negative monkeypox results. In this context, the aim of our study is to correctly classify the effect of monkeypox on skin lesions, which have started to spread rapidly on a global scale, by distinguishing it quickly and with a high accuracy rate through transfer learning methods.

The paper is divided into the following sections: Section 2 describes the related work of monkeypox disease along with a comparison to other methods. Section 3 describes the proposed methodology and proposed model structure through which the disease is evaluated. Section 4 describes the performance evaluation and experimental results, where a comparison is performed with other benchmark schemes. Section 5 discusses this study by comparing the results of previous studies. Finally, Section 6 concludes the paper.

## 2. Literature Survey

Past studies were examined within the scope of the classification of monkeypox disease with deep learning methods. Within the scope of the classification of monkeypox skin lesions with deep learning methods, researchers created skin lesion images from open-source websites and applied data augmentation methods with 3-k-fold cross-validation to increase the amount of data. The researchers tested pre-trained VGG-16, ResNet50 and InceptionV3 models to classify monkeypox and other diseases. Among these models, the ResNet50 was used as it has the highest accuracy rate. The ResNet50 has an F1 Score of 0.82 [4].

The Xception transfer learning model with Grad-Cam and LIME techniques has been used [5]. The Xception model and the DenseNet model have designed a community approach using unity. If the performance scores obtained from experiments on a publicly available dataset are analyzed in the study, which was completed with the help of the proposed ensemble approach, the average precision, recall, F1 score and accuracy was 85.44%, 85.47%, 85.40% and 87.13%.

Researchers [6] modalized the transfer learning method in the detection of monkeypox disease. They shared their own image dataset via GitHub. They created the dataset by collecting it from online websites. They proposed a modified VGG16 model involving two different studies and obtained AUC scores of 0.88–0.97 using the VGG16 model.

In another study, “Human Monkey Disease Classification from Skin Lesion Images with Pre-Trained Deep Mesh Using Mobile Application” [7] was aimed. In this context, classification was carried out with transfer learning methods over image data using Mobile Android application. The authors did not make any optimizations on the MobileNetv2 model. They used a dataset via the pre-published Kaggle. ResNet18, GoogleNet, EfficientNetb0, NasnetMobile, ShuffleNet, and MobileNetv2 models were used in the studies. The best score was achieved on the MobileNetv2 model, with an accuracy of 0.91 and an F1-score of 0.90.

Another study [8] used the ResNet50 architecture to explore convolutional neural networks with transfer learning to diagnose Lyme disease from skin lesion images, and as a result achieved scores of 0.91 AUC, 0.83 sensitivity, 0.87 accuracy, and 0.80 specificity. When convolutional neural networks (CNNs) and transfer learning methods are used together, high performance can be achieved in classification studies of skin lesion images.

In another study, conducted by [9] within the scope of the classification of skin lesions, classification was made through the study of automatic detection of erythema migrans and other skin lesions by deep learning methods in the detection of Lyme disease. As a result, an accuracy of 0.86 and AUC score of 0.95 were achieved with deep learning.

In this study, the aim was to distinguish monkeypox lesions from other similar skin disease lesions with high accuracy, sensitivity, and selectivity by using CNN and transfer learning methods. A comparison of past studies is shown in Table 1. The scores of the studies are given in the discussion section.

In previous approaches, it is seen that the dataset is collected jointly through the open-source web. When we started our study, there were no publications and datasets about monkeypox disease. As in other studies, the dataset was collected as open source from news and health institution websites. It is grouped by increasing and classifying data on Roboflow. As seen in other studies, although researchers using standard transfer learning methods used the same data, performance rates were limited to 0.80–0.90.

In our study, we used the latest version of transfer learning models, such as EfficientNetV2s, MobileNetV3, VGG19, ResNet50, DenseNet, and Xception. We have established a customized transfer learning model. We added intermediate layers and different activation functions to the model before the output layer. Within the same model, we used cross-layer hybrid activation functions (Relu and Softmax). The hyperparameters of our model are customized specifically for the installed model. In the training of the model, “Adam” was used instead of the “SDG” optimizer used in other studies. In our study, with hyperparameter optimization and customization of model layers, the highest accuracy rate, with an F1-score of 0.978, was achieved with the MobileNetV3-s model. Minor modifications to the hyperparameters of transfer learning models, customization of the models, and use of the hybrid activation function can achieve very high accuracy rates when used with the latest versions of transfer learning models.

## 3. Materials and Methods

Transfer learning is the use of deep learning methods to solve the problems they encounter by hiding the information they have learned in the past while solving a problem like in humans. By using transfer learning, it is possible to use a high-performance model with less data. Several CNN architectures have been proposed to improve relevant system performance by keeping the basic learning topology constant [10]. AlexNet, VGG16, VGG19, ResNet, DenseNet, MobileNet, and EfficientNet are the most frequently used CNN models for object recognition and classification tasks [11]. The use of CNN in deep learning is a machine learning method that has been studied most recently within the scope of SOTA.

### 3.1. Convolutional Neural Network (CNN)

**Figure 1:**
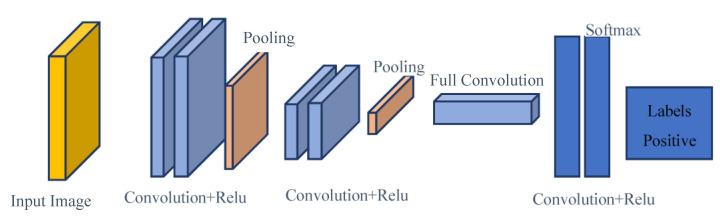
Basic CNN model architecture.

For medical images, structural information between neighboring pixels is an important source of information. Therefore, vectorization loses such structural information in images. A convolutional neural network (CNN) is designed to make better use of spatial and configuration information by taking 2D or 3D images on their own as input. Structurally, CNNs have convolution layers interspersed with pool layers, as in the standard many-tier neural network, followed by fully coupled layers [10]. The basic CNN architecture layers [12] are shown in Figure 1.

**Figure 2:**
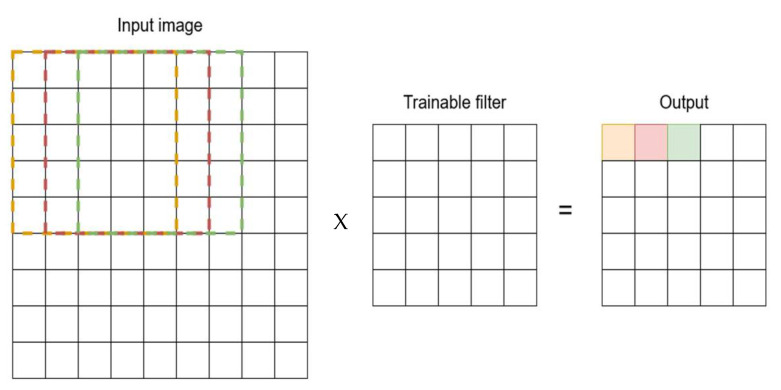
Convolutional layer.

CNNs are neural networks consisting of the convolutional layer and pool layers and can be fine-tuned with hyperparameters [13]. The convolution layer is used to create new multidimensional data from multidimensional data. As shown in Figure 2, the first entry image is 32 × 32 in size. The layer trains a 5 × 5 filter by going over each group of 5 × 5 pixels in the image and converting them to a single pixel in the output. The filter in the first step, instead of making a 32 × 32 entry, uses a 9 × 9 input. The convolution layer scans and uses the color (RGB) of each pixel in the input to create 6 output channels for each pixel in the output [14]. After the convolutional layer is run, the pooling layer’s structure allows the outputs to be compressed into smaller chunks.

**Figure 3:**
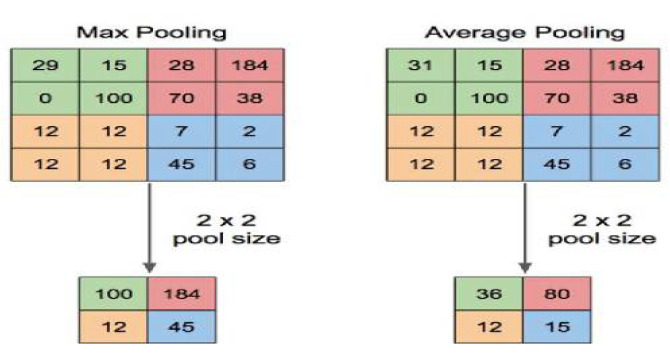
Pooling layers structure.

The outputs of the previous layer are compressed by the max or mean (avg.) function. The sample pooling layers structure [15] is shown in Figure 3.

**Figure 4:**
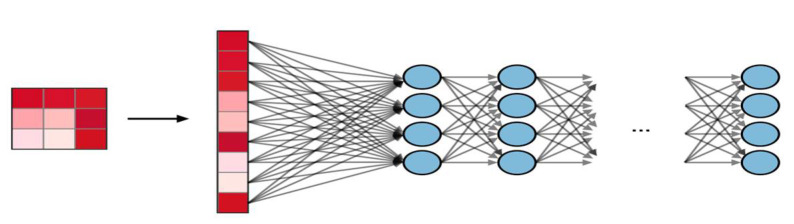
Full connection layer. Blue frames show hidden neurons.

The full connection (FC) layer operates at the input where each input is connected to all neurons. The FC layer often uses the last layer to optimize the classification results. The sample FC layer [15] is shown in Figure 4.

**Figure 5:**
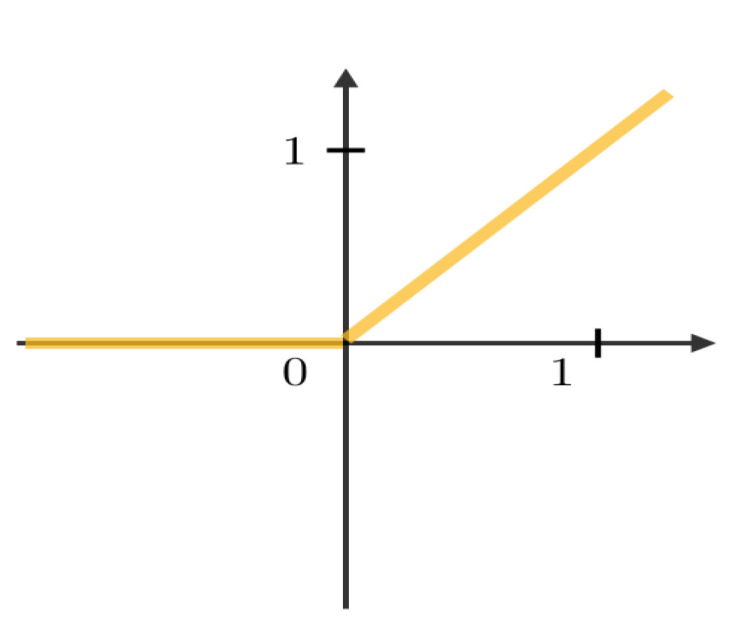
ReLU function chart.

The Relu activation function is the most common function used in hidden layers () [16]. The ReLU function graph we used in this study is shown in Figure 5 and Equation (1). The following are the Optimization Function Methods used in the evaluation of CNN models: Adam, Adamax, Nadam, and AdaDelta [17]. In this study, the Adam optimizer method was preferred. Adam is a first-order, gradient-based algorithm of stochastic purpose functions based on adaptive predictions of low-order moments. Adam is one of the state-of-the-art optimization algorithms used by many machine learning practitioners. The first moment, normalized by the second moment, gives the direction of the update [18].

g(z) = max(0,z) (1)

Adam’s update rule is shown in Equation (2).

(2) $$\theta_{n+1}=\theta_{n}-\frac{\alpha }{\sqrt{v_{n\;}+\epsilon }}m_{n}$$

The proposed Data Transformation Normalization study, which is a supervised model, consists of classified positive and negative monkeypox image samples. In or study, image samples need to be scaled in a digital format suitable for neural network input; in other words, normalized [19]. Our obtained data is between (0.255) and the RGB channel value. This range is not ideal for a neural network. For this reason, the values are normalized to the range (0–1).

### 3.2. Proposed Model Structure

**Figure 6:**
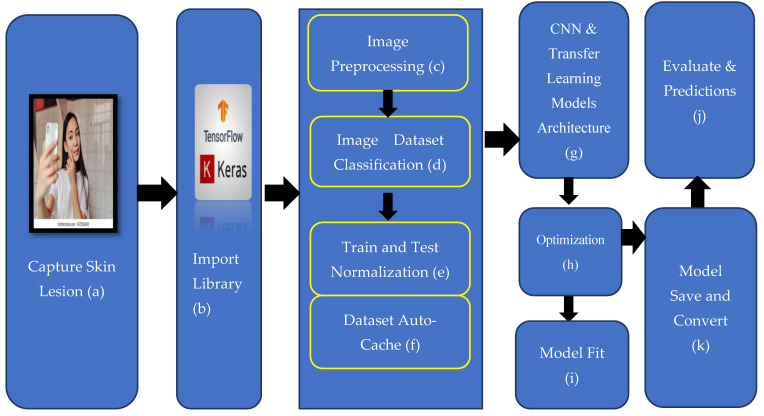
Architecture for classifying monkeypox skin lesions.

The general architectural design of our study is shown in Figure 6.

Step 1: Skin Capture with Mobile Camera

All users will be able to use this designed system easily. Therefore, the main input data source determined for our model are the skin images taken from mobile devices.

Step 2: Import Library

This will subject image data from mobile devices to pre-processing, normalization, and classification processes so that we can use them in our model. In this context, we have realized the transfer learning model and structural algorithms we will use by using the Keras and TensorFlow libraries. These libraries are the most commonly used libraries in image processing. TensorFlow was used to pre-process the images and the Keras library was used to design our transfer learning model.

Step 3: Image Pre-processing

We have to pre-process skin images from mobile devices before they can be trained or tested on our model. During the pre-processing phase, we ensure that the data received from the external source conforms to the format we have determined in our model. This stage is important both for training our model and for us to reach high accuracy scores during the testing phase. Our system starts with the import of the image taken from the external source into the system. Skin images are needed to be used both in the training phase and in the testing phase. During the test and application phase, the plan is to take the image via mobile devices (Phone, Tablet, and PDA) because the use of mobile devices will provide portability, easy data acquisition, and easy use by end users. At the end of the work, the user of any mobile device will be able to train or test this application. Mobile learning models require image data in a specific format. We have converted the image data we received from the external source into the format requested by the transfer learning model we will use. In this way, we have achieved high accuracy rates by making our model work more smoothly and efficiently.

Step 4: Image Dataset Classification

We had to separate the skin images that had passed the pre-processing stage as positive and negative before entering the training phase. We collected the positive monkeypox skin images in one folder and the negative images in a separate folder. Classification labels are determined based on these folders. During the pre-processing phase according to the classification labels, the training and test data labeling mode was determined categorically. We have separated the data we allocated for training as 30% validation data. As a result, we classified our data by ethnicking them according to 2 groups: positive and negative monkeypox.

Step 5: Train and Test Normalization

The process is optimized by using the cache, prefetch, and shuffle functions before training the training data and test data that we separated by tagging in the previous step. The buffer is designated as Autotune and a dynamic structure is proposed. Subsequently, a normalization layer was created. The normalization layer was defined to normalize the data received as an input between 0-1. We applied the map transformation only to the training data with the lambda function.

Step 6: Transfer Learning Models

The Transfer learning model enables models that have previously been trained with large amounts of data to be quickly introduced into training without being trained from scratch. There are many types of transfer learning models. The number of parameters, layer types and numbers, and depths differ from each other. We started the training by choosing the model that best suits our own design and data. When used in the default settings, the output layer is added to the transfer learning models, and the result is expected. We added an additional layer between the output layer and the flatten layer, which is suitable for the model we used in our study. We recalculated the output layer with the softmax function by defining the activation function of this layer as “ReLU” and connecting it to the Output layer. By using each softmax and Relu functions consecutively, positive results were obtained on model performance. EfficientNetV2s, MobileNetV3, VGG19, ResNet50 DenseNet, and Xception transfer learning models are trained on this model. We tried to get the best score by changing the hyperparameters.

Step 7: Model Optimization

After determining the transfer learning model that we will use in our design, the scores were observed by changing the hyperparameters of the selected model. Especially in transfer learning models, the selection of batch size, epoch, loss function, and optimizer directly affect the results. In particular, the epoch number should be kept at the optimum level against the overfitting and underfitting situations of the model. In fact, one of the biggest advantages of transfer learning models is that good scores can be achieved at low epoch numbers. We were able to improve the scores by adding interstitutions to our model. We tried Adam, AdamX, SGD, AdaGrad, AdaDelta, and RMSProp. The best score as optimizer was provided by the Adam function. The Stochastic Gradient Descent (SGD) optimization tool comes by default in the models; unfortunately, it cannot give successful results in image processing problems. For this reason, adaptive algorithms with dynamic structure were used instead of SGD. The Adaptive Momentum algorithm we used in our model stores the learning rates and momentum changes of each of the parameters in the cache.

Step 8: Model Fit and TensorBoard View Use

We include a training set and validation set in the model we prepared in the previous step.The epoch value is set to “20” and the batch size value is set to “64”. In order for us to monitor the results on TensorBoard, the callback parameter is added and our model with all parameters starts training with the “fit” method.

TensorBoard is a module included in the Keras library. With the callbacks method, it allows us to monitor the metrics of the real-time model during the training phase. In this way, it has allowed us to monitor instantly whether there is overfitting or underfitting. It allowed us to evaluate the training results visually by following the metrics in detail.

Step 9: Evaluate and Prediction

Following the completion of the training process of our model, the model must be verified and tested. With the evaluate process, we import the data we have pre-processed as a test dataset to evaluate. The results will give performance metrics on the test data of our trained model. We use the prediction method to test image data that the model has never learned before. To test the data in our model, we first pre-processed them and brought them to the appropriate size for our model. Subsequently, we converted our image data to the array type. We tested our transformed image data in our model with the test prediction method. According to the score, the proximity to 2 (positive and negative) classes is revealed as a percentage. The highest rate determines the classification.

Step 10: Model Save and Convert

We need to save our designed and trained model so that we can reuse it internally and externally in the work environment. When we save our model in standard settings, the tilted model size will be high, which makes it difficult to use on mobile devices. For this reason, we have converted our model to a format where mobile devices can be used with TFLite. In this way, the size of our skewed model file, which is approximately 30 MB in size, has decreased to 2.7 MB with TFLite.

In our study, EfficientNetV2s, MobileNetV3, Vgg19, ResNet50, and DenseNet models were optimized as CNN algorithms. The general information of our model running on the TensorFlow framework is described in Table 2. During the training phase, the Tesla T4 (15 GB) model GPU was used. The training time on the GPU was 22 min. Based on the two models with the best scores (EfficientNetV2s, MobileNetV3), the model data sheet is shown in Table 2.

**Figure 7:**
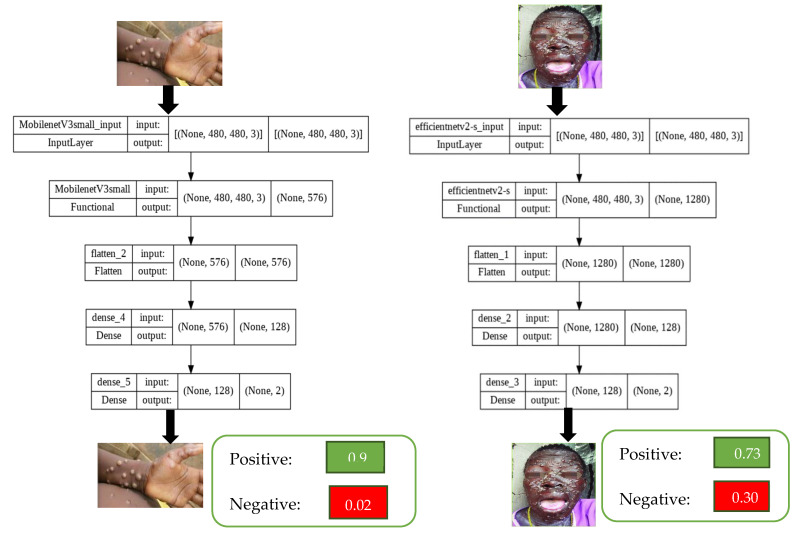
CNN and transfer learning model structure.

The optimized structural diagram of the EfficientNetV2s and MobileNetV3 transfer learning models is shown in Figure 7. Summary information about our transfer learning model is available in Table 2, including the size of the image, which is one of the most important parameters for our model. Knowledge of this parameter determines the input format of training and test data.

In Figure 7, we see the specialized structure of our transfer learning model. High performance could not be achieved with the standard parameters of the MobilnetV3Small model. That’s why we’ve customized our transfer learning model. As seen in Figure 7, it shows the transitions of the pre-processed image between the input and output layers in the transfer learning model. Here, the number of layers and scores of MobilNetV3-s and EfficientNetV2-s models are compared. In the default transfer learning model, the input layer consists of a flattening and an output layer. The output layer has to be as large as the number of our classes. Here, our output layer number is 2 (positive and negative). We set the number of intermediate layers we added at the optimum 128 and chose ReLU as our activation function. The input values of the next output layer are determined by the output of the intermediate layer. Therefore, the input of the output layer consists of 128 neurons and the output consists of 2 neurons. The softmax activation function was used in the output layer. In our model, “avg.” method was used as pooling. In this context, Figure 7 explains the input and exit parameters and ranking of the transfer learning method we have customized. In this way, the effect of making customizations in the transfer learning model design on the outputs is clearly stated. We can export the customized model structure with the plot_model method under the utilities class of the Keras library.

### 3.3. Dataset Acquisition

At the time we started our study, there was no currently published monkeypox image dataset. We scraped the image dataset that we use in our study as open source in two parts over the web. We have collected them in two different classes as positive and negative. Only image data of skin lesions belonging to monkeypox patients were added to the positive folder. In the negative folder, we included skin lesions from Lyme, Drug Rash, Pityriasis Rosea Rash, and Ring Worm diseases, which are similar to monkeypox skin lesions. In our dataset, a total of 2056 images were obtained with data augmentation methods. A total of 1742 image files from this dataset were used for training. In the training dataset, 1200 images contain positive lesions and 542 images contain negative lesions. For the test 156 images used, 90 of these images contained positive lesions and 66 images contained negative lesions. In obtaining the test data, the monkeypox skin on the Kaggle was used in the Lesion Dataset [20].

In this study, we created our dataset [21] as open source by dividing it into classes on the Roboflow platform. Base methods that do not disrupt the image structure were used to minimize data loss in the process of increasing the dataset of skin lesions. The methods used were Flip (Horizontal, Vertical), 90° Rotate (Clockwise, Counter-Clockwise, Upside Down). With the data augmentation method, our number of images has doubled. 

## 4. Performance Evaluation and Experimental Results

### 4.1. Parameters and Evaluation Metrics

In this study, the most commonly used transfer learning model is used among the deep learning methods. With the transfer learning model, deep learning can be done quickly by using a pre-trained and designed model structure. In this context, increasing the performance of the transfer learning model can be increased by updating hyperparameters.

Hyperparameters are parameters that vary across the model, dataset, and optimization models that are left to the designer of the deep learning model. In our deep learning model, the aim is to determine the optimum values of batch size, number of layers, number of neurons, dropout value, epoch number, split rate, normalization rate, learning rate, activation function, optimizer, and loss function selections. Changes made to hyperparameters, especially dropout and batch size values, were significantly altered in the results. The Stochastic Gradient Descent (SGD) optimization model did not produce successful results. Instead, thanks to the dynamic structure of adaptive algorithms, it is a great advantage that it learns the learning speed itself. The result of the loss function is targeted closest to zero. In our study, mean squared error (MSE) was used as the loss function. With the changes in the hyperparameters in the table below, the optimum score was reached in our study. The hyperparameter values used in this study are shown in Table 3.

Authors should discuss the results and how they can be interpreted from the perspective of previous studies and of the working hypotheses. The findings and their implications should be discussed in the broadest context possible. Future research directions may also be highlighted.

**Figure 8:**
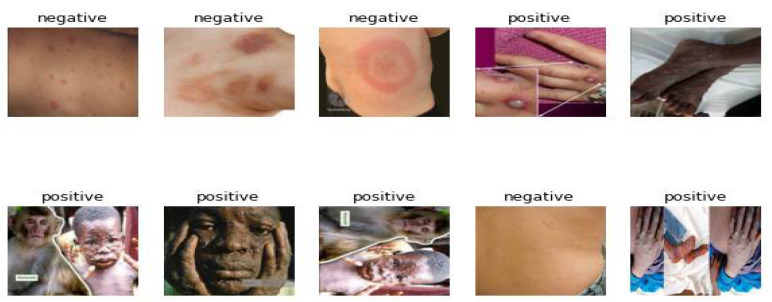
Sample views from the skin lesions dataset.

As shown in the dataset, we used 224 × 224 px monkeypox consisting of positive and negative pictures. The transfer learning model used in Figure 8 contains schematic information about the number of input layers, the number of output layers, and layer types. In Figure 8, the differences in the number of parameters of the two different transfer models and the scores we obtained in the output layer were compared.

With the web scrapping method, data was collected over the pages of public areas (hospitals, research centers, and global search engine). When it was evaluated that there was an insufficient amount of data in the image classification stages of monkeypox disease and that the supply would not be possible in a short time, the data augmentation method was used [22]. Apart from that, different data are collected from different databases—i.e., through cloud and big data [23,24,25,26]—and these data centers can be accessed wired and wirelessly. In the main dataset, there are 204 negative and 574 positive monkeypox lesion images as training data. With this method, the number of training data in our study reached 2056 images. Some 85% of the entire dataset is reserved as training and 15% as test data. The test data were selected from the data section, which had never participated in the validation parts of the model previously used in training and hyperparameter optimization.

There are different measurement criteria used to evaluate object detection model performance. In this study, and generally accepted in the literature, MSE, AUC, accuracy, loss function, and F1-score metrics were used [27].

Mean Squared Error (MSE): The learning model measures the performance of the predictor, which is always positive-valued and outperforms predictors with an MSE value close to zero [28]. The calculation of the MSE value is shown in Equation (3).

(3) $$\mathrm{MSE}=\frac{1}{n}\sum \begin{array}{l} n\\ j=1 \end{array}e_{j}^{2}$$

Area Under the Curve (AUC): The AUC value is used as an important metric in classification problems. The AUC value refers to the area under the ROC curve. A score close to 1.00 indicates that the classification is good. A score greater than 0.50 must be obtained for the model to be acceptable.

Accuracy: This shows how close a measurement is to a defined or accepted. The calculation of the accuracy value is shown in Equation (4).

(4) $$\mathrm{ACC}=\frac{\mathrm{TP}+\mathrm{TN}}{\mathrm{TP}+\mathrm{TN}+\mathrm{FP}+\mathrm{FN}}$$

Recall: This is the accuracy rate. Recall is the opposite of certainty; it calculates false negatives versus true positives. Recall is shown in Equation (5).

(5) $$\mathrm{Recall}=\frac{\mathrm{TP}}{\mathrm{TP}+\mathrm{FN}}$$

Precision: This is a positive analytic value. It defines how reliable the measurements are, even if they are far from the accepted value. The calculation of the precision value is shown in Equation (6).

(6) $$\mathrm{Precision}=\frac{\mathrm{TP}}{\mathrm{TP}+\mathrm{FP}}$$

F1-score: F1-score is used against the trade-off between recall and precision metrics. The harmonic mean is used instead of the F1-score arithmetic mean [29]. The method of calculating the score is shown in Equation (7).

(7) $$F_{1\left(\mathrm{Score}\right)}=2\;\frac{\mathrm{Precision}\;x\;\mathrm{Recall}}{\mathrm{Precision}+\mathrm{Recall}}$$

### 4.2. Result Discussion

**Figure 9:**
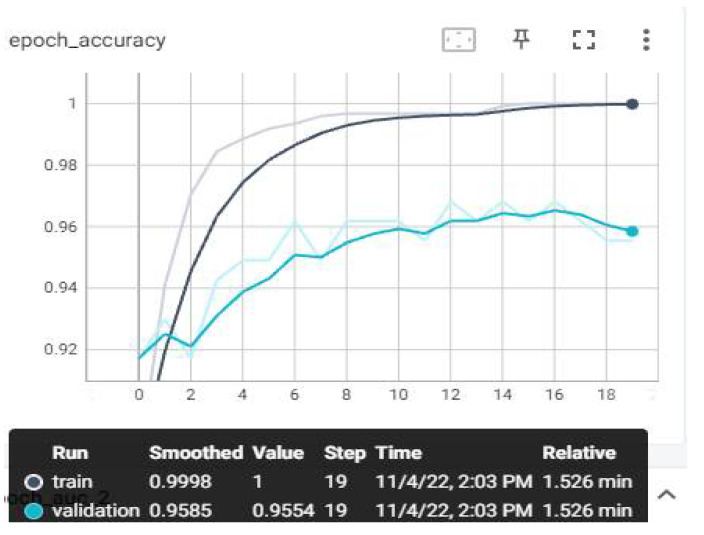
Experimental Result Accuracy—Epoch Graph.

**Figure 10:**
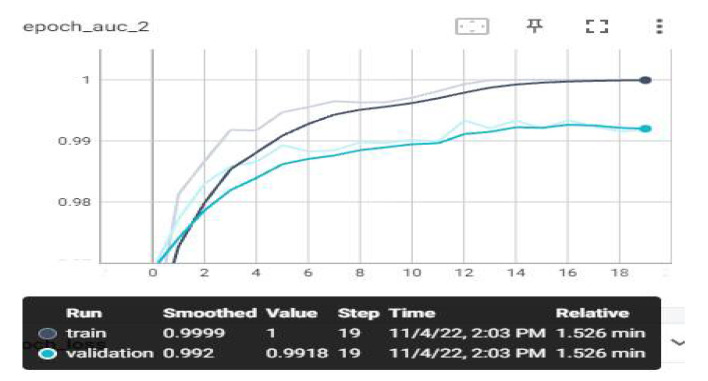
Experimental Result—AUC–Epoch Graph.

**Figure 11:**
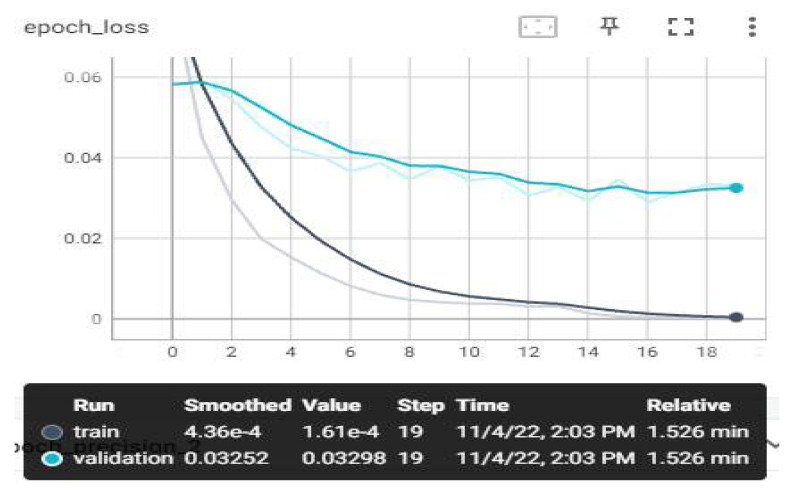
Experimental Result—Loss–Epoch Graph.

**Figure 12:**
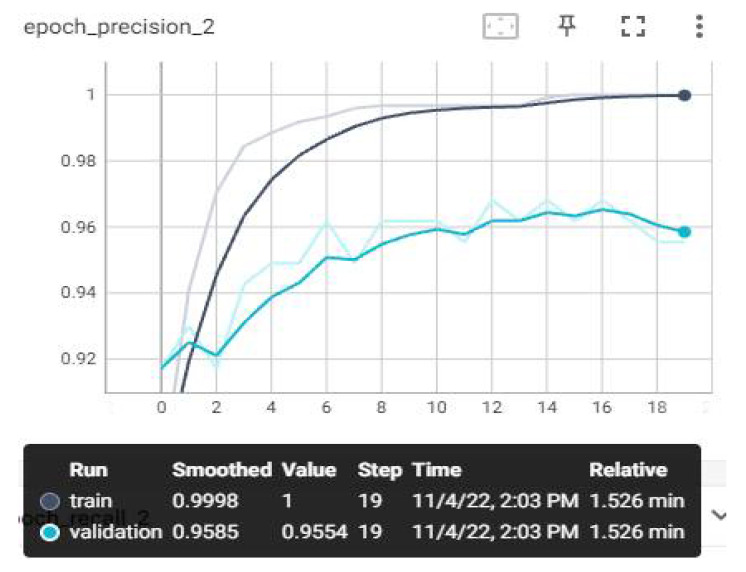
Experimental Result—Precision–Epoch Graph.

**Figure 13:**
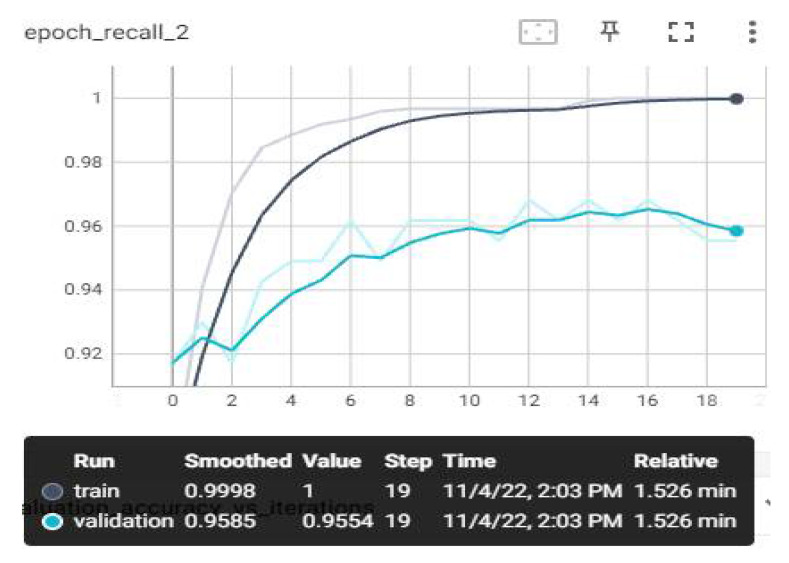
Experimental Result—Recall–Epoch Graph.

The loss and estimation values that we have monitored on TensorBoard after the training are shown below. Evaluation metrics involve accuracy, AUC, recall, precision, and loss graphs. From the obtained values, the F1-score value was also calculated using the calculation in Equation (7). The highest average F1-score value was achieved with the MobileNetV3 model, with 97.84%. The AUC, accuracy, loss, precision, and recall graphs are shown in Figure 9, Figure 10, Figure 11, Figure 12 and Figure 13.

In Figure 9, the accuracy evaluation graph is the ratio of accurate predictions to all predictions in our model. It is one of the easiest metrics to understand and interpret.

Machine learning is often used in classification problems [30]. Accuracy takes a value between 0 and 1. The closer the score is to 1, the more successful it is. When we examined our model, it completed the learning model in 20 steps (0–19) and obtained an accuracy score of 0.9998 in the training data. A score of 0.9554 was obtained in the verification data. When the graph is examined, overfitting or underfitting situations in which the curves progress stably did not occur.

In Figure 10, the AUC rating chart denotes the value below the ROC curve, which is between 0 and 1. The closer it is to the correct 1, the more successful the model is. When we examine our model, it is seen that the AUC value starts from 0.86 and reaches the value of 1. It scored closest to 1 in both training and testing data. When the model AUC curves were examined, the training and test data were stable, and underfitting and overfitting situations did not occur.

In the loss valuation chart in Figure 11, loss can be called a bad prediction penalty. If the model’s prediction is perfect, the loss is close to zero. The goal in training a model is to find the lowest losing weight. The main purpose of our study is to reduce the loss of the model to the point closest to zero. When the graph is examined, the loss in education data has reached a score of 0.000436. In the test data, it was reduced to a score of 0.0325. The scores came very close to zero. The loss score is considered successful. Underfitting and overfitting were not observed.

The values in Figure 12 are known as precision. It is considered successful in a positively predicted situation. The Graph in Figure 12 shows how many of the values considered positive are true positive. High precision value is an important criterion in model selection. When the graph was examined, a precision score was obtained in training data and a 0.998 score in test data. The fact that the accuracy value is high indicates that the model selection is correct.

The recall value in Figure 13 is a metric that indicates how much of the transactions we need to predict as positive are predicted as positive. It is also called sensitivity. It is desirable that the sensitivity value is high. When our graph is examined, a score of 0.999 was obtained in the training data.

The most advantageous aspect of transfer learning is the lack of long training periods. Our study took about 20 min on a Tesla T4 GPU in 20 epoch numbers. The MobileNet model averaged AUC scores of 99.7%, accuracy of 99.1%, recall of 99.1%, precision of 99.1%, and F1-score of 99.18% on the training dataset trained in a short time. The test dataset consisted of data that had not previously been included in the training dataset. The MobileNet model averaged AUC scores of 99%, accuracy of 96.8%, recall of 96.20%, precision of 96.8%, and F1-score of 96.50% on the test dataset. The loss value was scored on the training dataset (0.009) and on the validation dataset (0.03). The average scoreboard obtained from VGG19, Resnet, MobileNet, DenseNet, EfficientNetV2, and Xception transfer models is also shown in Table 4.

**Figure 14:**
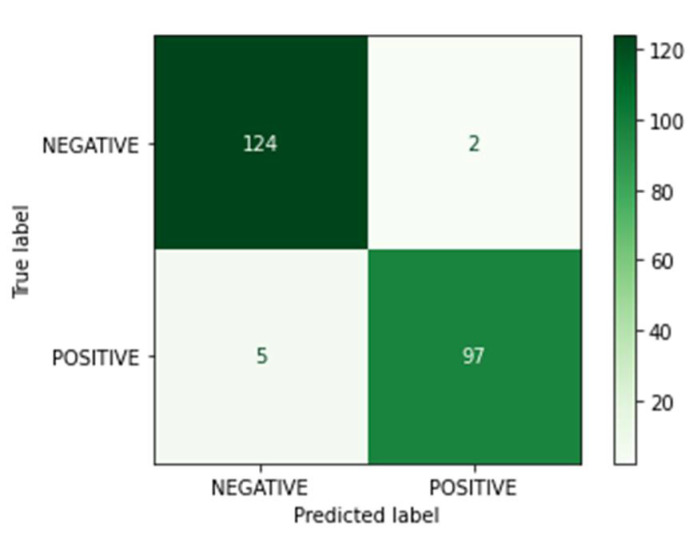
MobileNetV3 model confusion matrix.

The confusion matrix after the prediction we made with the MobileNet model on the test dataset is shown in Figure 14. The mean F1-score is 0.978.

**Figure 15:**
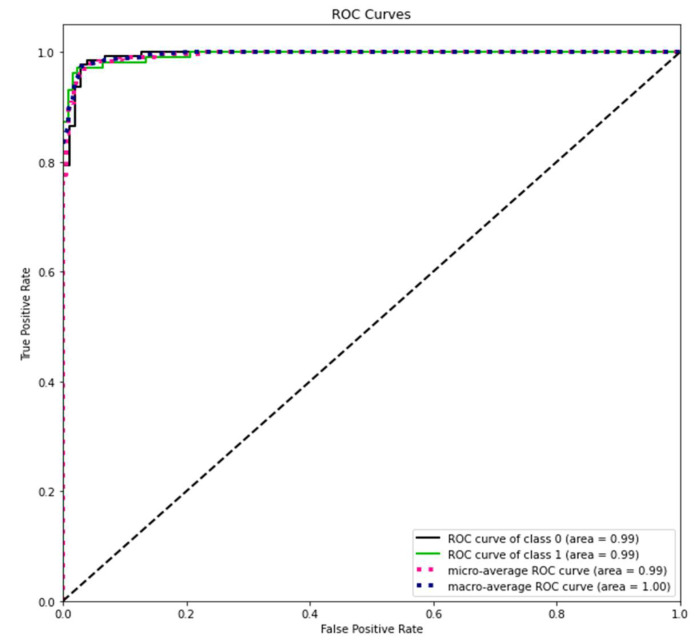
MobileNet model ROC curves.

The ROC curve we obtained after the prediction we made with the MobileNet model on the test dataset is shown in Figure 15. It can be seen from the ROC curve that the classification scores are the point closest to 1. It is an indication that our model is successful.

As seen in Table 4, when the evaluation metrics were examined, the transfer model with the highest F1-score value was determined as MobileNetV3-s. In our study, the conversion of the trained MobileNetV3-s model to a TensorFlow TFLite model that can work on mobile devices was also achieved. In this way, the working outputs are made suitable for the use of all humanity by working quickly on Android and IOS operating system devices without requiring special devices. The test and validation datasets consist of data that have not previously been trained. As can be seen, with the high accuracy rates of the correct hyperparameter and optimizers selection on the models, monkeypox can be classified as positive and negative over skin lesions.

## 5. Discussion

Table 5 presents a comparison of this study with previous studies. This study has tried to prove that the usability of image classification procedures in areas of public health can contribute to the shortening of the diagnosis and evaluation processes of diseases.

During the preliminary diagnosis of monkeypox disease, it was observed that it was common to confuse skin rashes with skin lesions of similar diseases. The most important feature of the CNN method, one of the deep learning methods [31,32,33], is that it can successfully infer attributes by passing the visuals through the convolutional layer and filters. When transfer learning models that have already been successfully trained with thousands of data points are used in conjunction with CNN, high accuracy rates can be achieved in fewer epoch numbers. However, it has been observed that overfitting occurs when the epoch number is kept high in the training of transfer learning. Especially in transfer learning methods, even very small changes in hyperparameters have enabled us to achieve high performance. In addition, the CNN model that we have optimized has been converted to TFLite model and used on mobile devices. In this way, it is valuable in terms of enabling mobile devices to be used actively in the diagnosis of monkeypox disease. When the experimental results were evaluated, the correct classification was obtained with 89–97.8% F1-score values by making the right parameter selections with different transfer learning methods in our study.

In our study, the highest accuracy rate with an F1-score of 97.8% was achieved with the MobileNetV3-s model. Very high accuracy rates are achieved with very small changes in hyperparameters in the transfer learning models. When other studies were examined, the datasets used for the detection of monkeypox disease consisted of images obtained as open source through Google Images; likewise with our study. Ali et al. [4] achieved an F1-score of 83% with the VGG16. Ahsan et al. [6] obtained an F1-score value of 83% on the test data with the VGG16 model. The reason why we have achieved high performance is the customized hyperparameter and optimization methods. For instance, the transfer learning model was used with the default hyperparameters, and scores of 80-85% were obtained by Ali et al. [4]. Our study also shows effectiveness of the selection of the correct hyperparameters (epoch, batch size, learning rate, image size, activation function, pooling) and optimizer selection in transfer learning.

Within the scope of the study, data were collected through the websites of public news and health organizations with the web scrapping method. Recent studies [4,6] obtained image data from the same methods and sources. With data replication methods, a sufficient number of image samples were obtained for deep learning. The reason for this is that high-resolution skin lesion images of monkeypox patients have not yet been published and it is considered that it will not be possible to provide them in a short time. For this reason, it is of great importance for international health institutions to create and make available monkeypox image datasets in terms of future studies. It will be especially valuable in terms of testing and comparing more stable, fast, and accurate models.

What sets our work apart from other studies and the reason why we have achieved high performance is the customized hyperparameter and optimization methods. When the transfer learning model was used with the default hyperparameters, scores of 80–85% by [4] were obtained. This study shows how effective the correct hyperparameters (epoch, batch size, learning rate, image size, activation function, pooling) and optimizer selection are in transfer learning. When past studies are examined, a high-resolution image dataset has not been created and published by an official health institution related to monkeypox. However, in the deep learning structure, we have seen how high the CNN transfer learning models can achieve a high level of achievement in classification even at low epoch numbers. Despite the low-quality dataset, the fact that we achieve classification performance with high accuracy increases our admiration for CNN structures. In image classification within the scope of SOTA studies, [5] mentioned LSTM and BERT algorithms, especially those used in natural language processing. However, these algorithms use the NLP field more than image processing. Another study treated urine sediment particle detection as an object detection task and proposed YOLOv5s-CBL, a detector dedicated to particle detection [34]. They compared state-of-the-art methods in a real-world dataset, and the experimental results demonstrated the efficacy of YOLOv5s-CBL. Another study [35] recommended the YOLOv5x-GCB model for the timely diagnosis of intracranial hemorrhage (SCC) by computed tomography (CT) imaging method. As a result of the study, intracranial bleeding detection was successfully realized with an F1-score of 0.90. Another study introduced a Detectron2 and Faster R-CNN to automatically diagnose COVID-19 from X-ray images, and also evaluated that this work could support non-radiologists with better localization of the disease with a visual bounding box [36]. For this reason, it would be more appropriate to use YOLO and Detectron2 structures in state-of-the-art (SOTA) image classification.

## 6. Conclusions

In the study, we aimed to accurately detect skin lesions with deep learning methods in the diagnosis of the monkeypox disease, which is spreading rapidly in the world. With the optimization and hyperparameter updates of the CNN and transfer learning models, high success and low loss rates were achieved. In this study, optimum values were investigated for the correct classification of hyperparameters, especially batch size, epoch, image size, layer count, activation function, optimizer, and loss function selections. Slight changes in the hyperparameters from the results obtained allow us to classify the results more accurately. The combination of optimum epoch with softmax activation functions in the ReLU and output layer between the hidden layers has led to high accuracy and excessive learning. Parameter values used are shown in Table 3.

In our study, transfer learning models (MobileNetV3-s, EfficientNetV2, ResNET50, VGG19, DenseNet121) with CNN were customized with hyperparameters and classified by differentiating monkeypox skin lesions from similar skin disease lesions. Although the similarity of the rashes in the skin lesions leads to errors and hesitations in the preliminary diagnosis stage, the highest accuracy rate, with an F1-score of 97.8%, was achieved with the MobileNetV3-s model. We think that transfer learning models can achieve very high accuracy rates with the right choices in hyperparameters. Training the model is crucial to solving all image processing problems, including the CNN models we used in our study. The cleaner the model can be trained with data, the healthier our results will be. Image datasets used to detect monkeypox disease are not classified by official medical professionals. There is no official dataset published by health institutions yet. The researchers scraped images from the web with their own CABAS. Future studies will be very valuable if official health institutions publish data on monkeypox skin lesions. In addition, we assess that high-accuracy results can be obtained by using YOLO and Detectron2 deep learning models in the classification of skin lesions.

## Figures and Tables

**Table 1:** Comparison of methods.

Author/Year	Purpose	Proposed Methodology	Key Parameters	Model
Ali et al., 2022 [4]	Monkeypox skin lesion detection	Monkeypox skin lesion detection using deep learning models	F1-score	VGG-16, ResNet50, and InceptionV3 models
Situla and Sahahi, 2022 [5]	Monkeypox virus detection	Detection of monkeypox virus by transfer learning methods	Accuracy and F1-score	Xception, DenseNet
Ahsan et al., 2020 [6]	Detecting monkeypox disease	Image data collection and implementation of a deep learning-based model in detecting monkeypox disease	AUC	They propose and evaluate a VGG16 model with D curve.
Sahin et al., 2022 [7]	Human monkeypox classification from skin lesion images	Human monkeypox classification from skin lesion images with deep pre-trained network	Accuracy and F1-score	GoogleNet, EfficientNetb0, NasnetMobile, ShuffleNet, MobileNetv2 models
Hossain et al., 2022 [8]	Lyme disease from skin lesion images	Convolutional neural networks with transfer learning to diagnose Lyme disease from skin lesion	AUC, sensitivity, accuracy and specificity	ResNet50
Philippe et al., 2019 [9]	Automated detection of erythema migrans	Automated detection of erythema migrans and other confounding skin lesions via deep learning	AUC and accuracy	Resnet50
Proposed Method	Monkeypox detection	Monkeypox detection using CNN with transfer learning	Accuracy and F1-score	EfficientNetV2s, MobileNetV3, VGG19, ResNet50, DenseNet

**Table 2:** Model Information.

Input Size	Model Parameters	Language	Environment	Framework, Library	Epoch	Optimizer	Activation Function
224 × 224	1,013,234	Python	Colab	TensorFlow, Keras	20	Adam	RELU & Softmax

**Table 3:** Hyper Parameters.

Parameters	Values
Image size	224 × 224
Batch size	20
Epoch number	64
Pre-processing validation split	0.3
Class number	2
Rescaling	1./255
Pooling	Avg
Weights	Imagenet
Layer Dense Activation Function	ReLU and Softmax
Total params	1,013,234
Optimizer	Adam
Loss Function	MSE
Image Dataset Size	2056

**Table 4:** Transfer learning models scoreboard.

Transfer Models		Average Values				
		AUC	Accuracy	Recall	Loss	F1-Score
MobileNetV3-s	Train	0.997	0.991	0.991	0.009	0.978
	Test (Uniq)	0.990	0.968	0.962	0.034	
EfficientNetV2	Train	0.997	0.992	0.992	0.006	0.973
	Test (Uniq)	0.989	0.955	0.955	0.034	
ResNET50	Train	0.992	0.981	0.981	0.015	0.958
	Test (Uniq)	0.988	0.935	0.935	0.047	
VGG19	Train	0.981	0.968	0.968	0.027	0.946
	Test (Uniq)	0.971	0.924	0.924	0.062	
DenseNet121	Train	0.926	0.896	0.896	0.093	0.895
	Test (Uniq)	0.927	0.895	0.895	0.096	
Xception	Train	0.851	0.862	0.862	0.140	0.866
	Test (Uniq)	0.842	0.852	0.852	0.144	

**Table 5:** Comparison with other studies.

Studies	Dataset	Model	F1-Score
Ali et al. [4]	Custom Dataset	VGG16	0.83
Sitaula [5]	Custom Dataset	Xception	0.86
Ahsan et al. [6]	Custom Dataset	VGG16	0.83
Şahin et al. [7]	Custom Dataset	MobileNetV2	0.90
Our Study	Custom Dataset	MobileNetV3-s	0.97

## Data Availability

The data can be provided when required.
